# Perceptions and Willingness of Patients and Caregivers on the Utilization of Patient-Generated Health Data: A Cross-Sectional Survey

**DOI:** 10.3390/ijerph22071099

**Published:** 2025-07-11

**Authors:** Ye-Eun Park, Sang Sook Beck, Yura Lee

**Affiliations:** 1 Department of Information Medicine, Asan Medical Center, College of Medicine, University of Ulsan, Seoul 05505, Republic of Korea; ra02554@amc.seoul.kr; 2 Asian Institute for Bioethics and Health Law, General Complex B/D Yonsei University Health System, Seoul 03722, Republic of Korea; beck@yuhs.ac

**Keywords:** patient-generated health data, information management, telemedicine, patient-centered care, personal health records

## Abstract

Patient-generated health data (PGHD) enhance traditional healthcare by enabling continuous monitoring and supporting personalized care, yet concerns over privacy, security, and integration into existing systems hinder broader adoption. This study examined the perceptions, awareness, and concerns of patients and caregivers regarding PGHD and assessed their willingness to share such data for clinical, research, and commercial purposes. A cross-sectional survey was conducted from 6 to 12 November 2023, involving 400 individuals with experience using PGHD. Participants completed structured questionnaires addressing health information management, PGHD usage, and attitudes toward its application. PGHD was most commonly used by patients with chronic conditions and guardians of minors, with tethered personal health record apps frequently utilized. Respondents identified improved self-management and better access to information as key benefits. However, significant concerns about data privacy and security emerged, especially regarding non-clinical use. Younger adults, particularly those in their 20s, showed lower willingness to engage with PGHD due to heightened privacy concerns. These findings suggest that, while support for clinical use of PGHD is strong, barriers related to trust and consent remain. Addressing privacy concerns and simplifying consent processes will be essential to promote equitable and responsible PGHD utilization across diverse patient populations.

## 1. Introduction

Patient-generated health data (PGHD), defined as data that are created, recorded, or gathered by or from patients, family members, or other caregivers to address a health concern, ref. [1] provide unique benefits beyond traditional healthcare data. PGHD aid patients by facilitating continuous care, monitoring, and management of high-risk patient groups [2]. The scope of PGHD includes patient-reported outcome measures (PROMs) in clinical trials and various lifestyle-related health choices; thus, the utilization of PGHD can help bridge information gaps between medical visits, reduce healthcare costs, and improve outcomes [3]. Given that PGHD incorporate patient environments and health-related behaviors, they provide a better reflection of the outcomes of real-world clinical intervention [3,4]. Notably, PGHD can enhance health outcomes and support clinical decision-making by providing a deeper understanding of patient needs through caregiver and patient engagement [5,6,7,8,9,10]. Nevertheless, challenges related to data protection, privacy, and integration hinder the utilization of PGHD [11,12,13,14]. Ensuring security and system compatibility plays an essential role in preventing breaches and addressing concerns regarding the quality and usability of data [3,15,16]. Addressing these issues is the key to fostering patient and caregiver trust and facilitating the widespread adoption of PGHD. Personal health records (PHRs), which are often used to manage PGHD, may be tethered (linked to institutional electronic health records) or untethered (commercial, standalone applications). Understanding how patients and caregivers engage with different PHR types is vital to supporting real-world PGHD adoption.

Understanding patient and caregiver perceptions plays a crucial role in facilitating meaningful, patient-centered PGHD utilization. The sharing of clinically relevant information, including PGHD, has been closely linked to patient engagement, which serves as a catalyst for improved treatment adherence, patient safety, and overall health outcomes. Therefore, this study explored the awareness and perceptions of PGHD among patients and caregivers, which are essential for driving patient participation in PGHD and achieving its full potential in healthcare.

## 2. Materials and Methods

A cross-sectional survey was conducted to assess the expectations and concerns of patients and caregivers regarding the utilization of patient-generated health data (PGHD). In this study, PGHD was defined as encompassing both clinical (e.g., treatment decisions) and lifestyle (e.g., daily activity) domains, regardless of the method of data collection. Structured questionnaires containing open-ended questions were used to examine perceptions of PGHD in both primary (clinical decision-making) and secondary (research, development, and commercial) contexts. In addition, variations in the generation and use of PGHD were explored according to the characteristics of patients and caregivers.

### 2.1. Questionnaire Development and Survey Design

The questionnaire comprised items assessing general respondent information, including the type of disease and health-related role; experience with health information management services; willingness to consent to the use of PGHD for medical purposes; willingness to consent to the use of PGHD for research or commercial purposes; and preferred participatory processes (Table 1).

The questionnaire was developed through expert consultation, involving a medical regulatory/legal expert (SSB) and medical informatics experts (YL and YEP). Further refinements were made in collaboration with specialists in PGHD research (SY), health IT (SYS), and medical information-law and ethics (KY) [4,13,17,18,19,20,21].

The responses to the survey items were collected using a combination of a 4-point Likert scale (ranging from 1 = Strongly Disagree to 4 = Strongly Agree); Yes/No responses; and single-choice, multiple-choice, and open-ended questions. The complete survey instrument, including all items and response options, is provided in Supplementary Materials (Table S1) for reference and transparency.

### 2.2. Participant Recruitment

**Figure 1:**
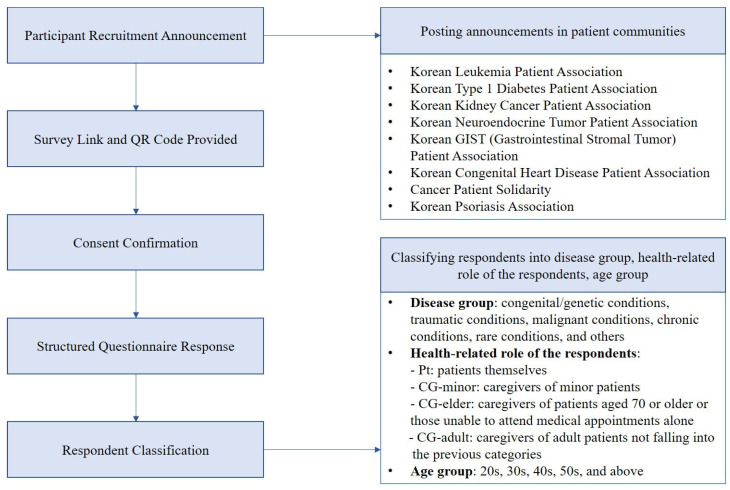
Recruitment process and participant distribution according to the type of disease.

Patients and caregivers with experience or potential engagement with PGHD were recruited to participate in the survey conducted from 6 November to 12 November 2023. Participants belonging to online patient communities related to leukemia, type 1 diabetes, kidney cancer, neuroendocrine tumors, GIST, congenital heart disease, cancer solidarity, and psoriasis were recruited to participate in the survey through public online announcements containing a survey link and QR code. Only individuals who provided informed consent were included in the survey. A minimum of 100 respondents were included from each major disease category (chronic diseases, malignant diseases, and other conditions) to ensure subgroup analysis (Figure 1).

### 2.3. Statistical Analysis

The respondents were categorized according to the type of disease, health-related role, and age group for subgroup analysis (Figure 1). The differences in categorical variables across ≥3 subgroups were assessed using the Kruskal–Wallis test. Continuous variables, including Likert scale responses, were analyzed using one-way ANOVA. The homogeneity of variances was assessed using Bartlett’s test. Statistical significance was set at *p* < 0.05. All data analyses were conducted using R (version 4.3.0).

## 3. Results

A total of 400 participants were included in the final analysis following the removal of four duplicate responses. Analysis of sex distribution across health-related roles (Table 2) revealed that the highest proportion of male participants was observed in the caregivers of older adults (CG-elder) group (43.2%). The highest percentages of patients in the 20s and >50 categories were observed in the patients themselves (Pt) group (** *p* < 0.01). The prevalence of chronic diseases was higher in the Pt, caregivers of minor patients (CG-minor), and CG-elder groups (Pt: 40.3%, CG-minor: 60.9%, CG-elder: 44.6%); in contrast, the prevalence of malignant diseases was higher in the caregivers of adult patients not falling into the other categories (CG-adult) group (56.6%). This difference was significant (** *p* < 0.01).

Significant differences were observed across different survey items for each group (Table 3 and Table 4). The Supplementary Materials (Tables S2–S4) present detailed analyses.

### 3.1. Experience with Health Information Management Services

Compared with the respondents in the other groups (Congenital/Genetic: 8.2%, 4/49; Malignant: 13.2%, 16/121; Chronic: 11.2%, 19/170), respondents with a traumatic condition were more likely to not manage their health information (24.1%, 14/58). Basic functions of mobile devices (37.8%, 151/400) were frequently used for the management of health information. However, respondents with chronic disease favored the use of disease management apps (41.8%, 71/170).

Notably, 78.8% (134/170) of respondents with chronic conditions reported using commercial PHR apps (i.e., smartphone applications or devices). In contrast, only 37.9% (22/58) of the respondents in the traumatic conditions group reported using commercial PHR apps. The most frequent measurements tracked among the 247 users were physical activity (N = 178), blood sugar levels (N = 114), heart rate (N = 106), and blood pressure (N = 85). The highest usage was observed in the CG-minor group (75%, 69/92), followed by the Pt group (66.9%, 121/181). The highest and lowest experience were observed in 40s (70.6%, 120/170) and 20s (52.4%, 22/42) age groups, respectively.

### 3.2. Tethered PHR Use

The highest usage of patient apps provided by medical institutions (24%, 29/121) was reported by respondents with malignant conditions, followed by those with traumatic (27%, 10/58) and chronic (11.8%, 20/170) conditions. The highest usage rate was observed in the Pt group (20.4%, 37/181), followed by the CG-minor group (18.5%, 17/92). Health information was accessed by 65.1% (41/63) of the respondents who used tethered PHR, and the primary benefit was self-management (63.4%, 26/41).

In terms of the duration of PHR app usage, 63.6% (14/22) of the respondents with traumatic conditions used the app for <6 months. A similar pattern was also observed among the respondents in their 30s (49.2%, 34/69). In terms of update frequency, the respondents in the Pt (52.1%, 63/121) and CG-minor (63.8%, 44/69) groups reported recording daily updates. Common reasons provided for discontinuing the use of PGHD apps were inconvenience (bothersome) (43.1%, 69/160), difficulty in use (30%, 48/160), and lack of health (management) benefits (22.5%, 36/160).

The respondents in the CG-minor group (86.8%, 65/75) exhibited the highest willingness to use tethered PHR. In contrast, the respondents in the 20s group were least willing to use tethered PHR (60.5%, 23/38). The respondents aged ≥40s expressed strong intentions to use tethered PHR, with rates exceeding 70% (40s: 81.9%, 118/170; >50: 73.9%, 34/58).

### 3.3. Attitudes Toward PGHD Use

**Figure 2:**
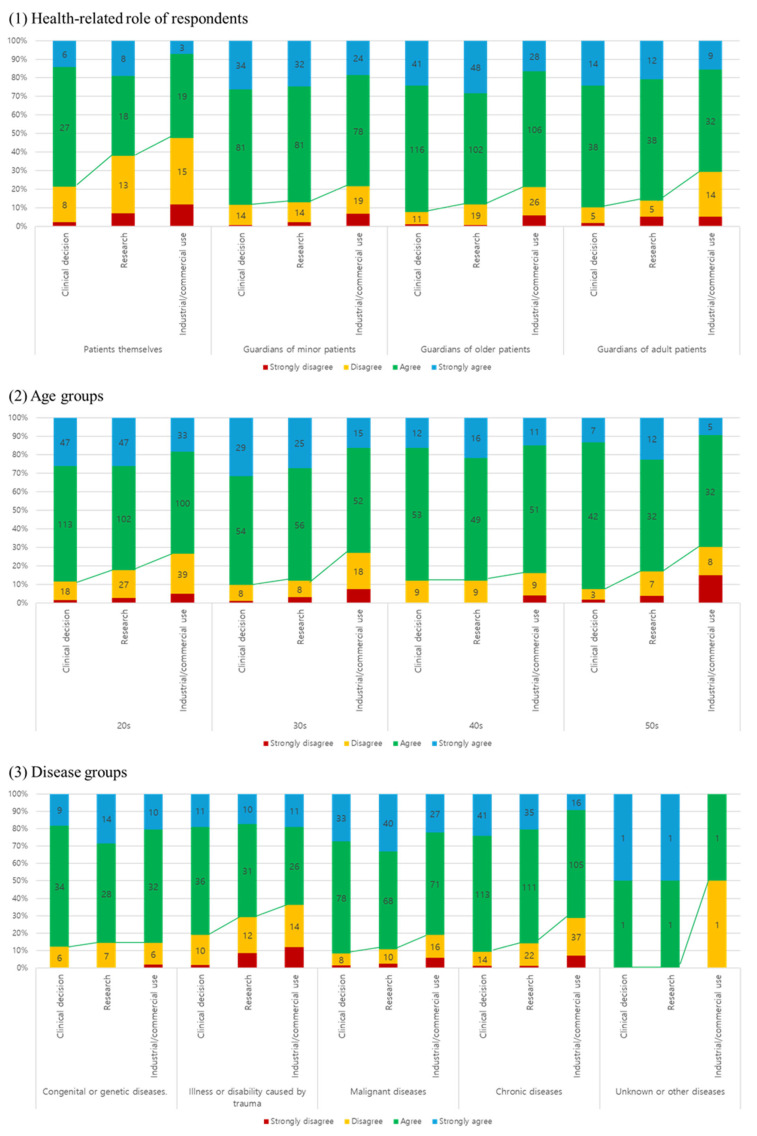
Attitude toward the use of patient-generated health data (PGHD) in accordance with the utilization purpose across the (**1**) disease-, (**2**) health-related role, and (**3**) age groups.

The respondents expressed strong support for the use of PGHD across clinical (89.3%, 357/400), research (84.8%, 339/400), and commercial/industrial (74.8%, 299/400) purposes (Figure 2). Personal information leakage was identified as the primary concern for research and commercial use (47.5% and 62.7%, respectively).

The highest willingness for comprehensive data usage was observed in the CG-minor group (58%, 47/92). In contrast, the use of anonymous data was favored by the CG-adult group (72.1%, 31/53).

The opinions of the respondents regarding whether they preferred consenting to certain studies on an individual basis (consent by specific research purpose) or providing a blanket agreement that would require no further permission requests (comprehensive consent) were obtained. The majority of the respondents with malignant conditions (52.9%, 64/121) favored providing comprehensive consent. In terms of health-related roles, the respondents in the CG-elder group exhibited the highest inclination towards providing comprehensive consent (55.4%, 41/74).

### 3.4. Responses on the Consent for Secondary Use

Table 5 presents the five most frequently reported reasons for selecting specific or comprehensive consent for the use of PGHD in research. In terms of specific consent, the respondents most frequently cited the desire to know the research purposes, contents, and duration, followed by concerns regarding the use of personal information and privacy breaches. In terms of comprehensive consent, convenience, the belief that it would help research, and time-saving were reported as the most common reasons.

The respondents prioritized the following in terms of the most important information for consenting to the use of PGHD: results of data analysis, research results, and implications (41.5%, 166/400); data protection and security issues related to data utilization (25.8%, 103/400); contact information for the responsible person (17.5%, 70/400); withdrawal process (14.5%, 58/400); and other factors (0.7%, 3/400). The respondents in all groups, except for those in their 20s and those with traumatic conditions, prioritized the results of PGHD. The respondents in their 20s and those with traumatic conditions prioritized data protection and security issues.

### 3.5. Other Sub-Responses and Responses to Open-Ended Questions

Table 6 presents various additional responses and feedback from the respondents regarding the methods used by them to manage or access health information. The use of photo-centric social networking services (SNSs) such as blogs and KakaoStory [22], as well as devices for monitoring blood glucose levels, was reported by the respondents.

In terms of the best aspects of using tethered PHR for managing health information, several respondents indicated that compared with the use of printed documents, the use of tethered PHR facilitated the review of the test results. Furthermore, the direct access to test results provided by tethered PHR, which did not require printed documents or visiting the hospital, was highly valued. Several respondents reported that reviewing test results in advance enabled better understanding and facilitated more in-depth conversations with healthcare providers.

In terms of frustrations with patient apps, respondents favored the accumulation of test results over a longer period. Some respondents noted confusion caused by unclear or inaccurate information, highlighting the requirement for more user-friendly language.

## 4. Discussion

This cross-sectional survey revealed significant differences in the patient or caregiver patterns of health information management and attitudes toward PGHD usage in terms of health-related roles, type of disease, and age group.

### 4.1. Principal Results

Differences were observed among respondent groups based on health-related roles (patients vs. guardians of minor patients), age, and sex distribution. These groups also varied in their experience and frequency of PGHD recording. Patients and guardians of minors (Pt and CG-minor) showed higher rates of PGHD recording and daily use compared to guardians of elderly or other adult patients. Overall, PGHD use was supported by a majority of respondents (average agreement rate of 82.9%), with concerns about personal information leakage being the most common reason for disagreement.

Despite these findings, the health-role-specific characteristics of PGHD use remain unclear. In a previous study, older users (mean age 51.81 vs. 43.81 years) were more likely to use PGHD functions continuously [23]; however, the study did not distinguish between older adults using the services themselves and guardians recording data on their behalf.

In this study, PGHD recording experience significantly differed by age and disease (** *p* < 0.01). The highest usage rate was found among patients with chronic diseases (78.8%, 134/170), followed by guardians of minors (75.0%, 69/92). These patterns suggest a greater need for ongoing health monitoring and active involvement in care among these groups. Therefore, beyond clinical needs, health-related roles may also influence the active use of PGHD.

The significance of this study lies in its distinction between PGHD users who were patients and those who were guardians, enabling a more nuanced analysis. Notable differences emerged in the use of patient apps provided by healthcare institutions, with the CG-minor group showing a higher willingness to use these apps. This group was also more likely to consent to the use of identifiable data for comprehensive, loss-free data utilization, whereas guardians in other groups preferred the use of anonymized data.

Survey results showed that among respondents who answered “strongly agree” to PGHD use, more supported its use for research (N = 100) than for clinical purposes (N = 95). Despite being a secondary use, PGHD for research was actively supported, particularly in the context of cancer and rare diseases. Respondents with malignant conditions showed the highest rate of strong agreement for research use (33.1%, 40/121). In the congenital/genetic disease group, support for research use (28.6%, 14/49) exceeded that for clinical use (18.4%, 9/49) by 10.2%. Support for PGHD in research was especially strong among those with malignant diseases, suggesting a greater interest in advancing scientific knowledge or perceived benefits from research-informed care.

This study identified age-related disparities in PGHD use. Compared to respondents in their 40s and 50s, younger individuals (20s and 30s) reported lower rates of PGHD recording. Reasons for discontinuing health management app use varied by age: younger respondents cited annoyance, while older users pointed to usability difficulties. Younger participants also showed lower intention to use such services and were less supportive of PGHD use for research purposes. Among those in their 20s, data protection and security were the most sought-after information when consenting to PGHD use.

These findings align with prior research indicating stronger concerns among younger users regarding mobile health (mHealth) services [24], consistent with the broader understanding that demographic characteristics influence behavioral intentions [25]. However, age-specific patterns in PGHD use remain underexplored. Collectively, this and previous studies highlight differences in attitudes and experiences with health data recording and management across age groups, underscoring the need to tailor engagement strategies to each demographic to promote inclusive participation in healthcare.

The most common reason for selecting specific (individual) consent for secondary PGHD use was a desire for clearer understanding of the process. The top responses—“want to know specific research purposes, contents, duration” and “want to know how personal information is used in research”—emphasize the importance of transparency and communication. In contrast, “convenience” was the primary reason for selecting comprehensive consent, suggesting that both informed understanding and procedural simplicity are key factors in user engagement. These findings are critical for designing systems that effectively obtain consent for the secondary use of PGHD.

In South Korea, while the Medical Service Act does not explicitly grant a right to data portability, patients can access their medical records, thereby enabling data portability with their consent. Recent amendments to the Personal Information Protection Act—specifically Article 35-2, enacted on 14 March 2023—formally grant individuals the right to request data transmission, aligning with the patient-centric data rights framework of the EU General Data Protection Regulation (GDPR). This amendment represents a significant step toward enhancing patient autonomy and underscores the need for further revisions to the Medical Service Act to more effectively support data portability in practice. Moreover, the finding that patients and their caregivers differ in their perceptions of PGHD use and their willingness to consent to secondary use has important implications for policymakers.

Despite Korea’s advanced medical information infrastructure and highly trained workforce, current legal provisions remain insufficient to ensure that requests for medical information transmission are patient-centered. In contrast, the United States enforces interoperability and information blocking rules under the HITECH Act, with HIPAA safeguarding medical transmission requests—though its data-sharing efforts are primarily coordinated at the regional rather than national level. To facilitate practical and differentiated policy decisions to leverage PGHD that can be linked to clinical practice, it is necessary to understand that patient and caregiver populations also have heterogeneity.

### 4.2. Limitations

The biggest limitation of the present study is that it was an online survey that targeted individuals belonging to the patient community. This population may not represent the general patient and caregiver population owing to potential bias in interest or engagement. Online surveys are limited; however, participants are more likely to have easy access to the internet. Furthermore, individuals belonging to the patient community are likely to have more active tendencies than patients or guardians who do not [26]. The 2016 National Statistics of Korea revealed that the median outpatient age was 50–54 years old, with approximately one-third (31.6%) of the participants being aged ≥65 years [27]. The age group with the most respondents in the present survey was the 40s (N = 170), with those aged ≥50 years accounting for 14.5% (58/400) of the total respondents. This is lower than the age distribution estimated in 2016. The inability of pediatric/older patients to directly respond was another limitation. Notwithstanding these drawbacks, the respondents were likely to have high accessibility to online information and make decisions regarding the use of PGHD. Hence, although bias in the selection of respondents was inevitable, the results of the present study better reflect the real world. Moreover, the female participants were proportionally overrepresented in the CG-minor group; this gender disparity potentially limits the broader applicability of our findings concerning gender-specific perspectives. Consequently, our results might not comprehensively reflect the subtle distinctions in attitudes toward caregiving responsibilities and PGHD interaction. Further investigative studies are recommended to examine gender-based variations in these areas using more balanced participant sampling.

The present study was based on the findings of a survey conducted only in Korea; thus, the results may not be generalizable. However, Korea exhibits a high level of development and maturity in terms of the use of EMR. Furthermore, citizens have high accessibility to online information. Thus, the use of this cohort facilitated a meaningful start to this discussion [28,29]. A national project for patient-centered health data utilization is being promoted in Korea. Consequently, confirming the awareness regarding the utilization of PGHD and its implementation in the establishment of systems and processes is necessary [30]. The present study confirmed patient awareness of PGHD usage and utilization; however, it was limited by its cross-sectional design. Awareness can change as the health status worsens or improves, and the health-related roles are also dynamic over time. These findings indicate the limitation of the questionnaire in its ability to address patient expectations and concerns. Technological advancements and policy changes can also affect patient awareness or attitudes towards PGHD. Thus, a continuous, multidisciplinary approach must be developed, and further research must be conducted in the future.

As with most survey-based studies, responses may have been affected by recall or acquiescence bias, particularly in Likert-scale items. Additionally, the inherent limitations of survey methods hindered the collection of in-depth participant insights, warranting further research.

## 5. Conclusions

This cross-sectional survey examined the differences in patient or caregiver patterns, attitudes, and anxiety towards PGHD management and usage according to their life situation. Most patient and caregiver respondents favored the use of PGHD; however, concerns regarding the security of personal information was reported. The respondents in their 20s and those with traumatic conditions were the most reluctant to use PGHD largely owing to privacy concerns.

## Figures and Tables

**Table 1:** Categories used in the finalized questionnaire.

Categories
A. General information, including type of disease and health-related (care-related) role of respondent (patient/caregiver)
B. Experience with health information management services with or without connection to the electronic health record (EHR) of the institution
C. Willingness to consent to the primary (medical) purposes of using patient-generated health data (PGHD) within the medical institution
D. Willingness to consent to secondary (research and commercial) uses of PGHD
E. Participatory processes that the respondents consider most important or are most curious about

**Table 2:** Demographic and health characteristic comparisons across the health-related roles of the respondent groups (Survey category A1–A4).

Variables	Pt (N = 181, 45.3%)	CG-Minor (N = 92, 23%)	CG-Elder (N = 74, 18.5%)	CG-Adult (N = 53, 13.3%)	*p*-Value	Total (N = 400)
**Sex**					0.04	
**Male**	64 (35.4%)	21 (22.8%)	32 (43.2%)	17 (32.1%)		134 (33.5%)
**Female**	117 (64.6%)	71 (77.2%)	42(56.8%)	36 (67.9%)		266 (66.5%)
**Age**					*p* < 0.01	
**20s**	30 (16.6%)	3 (3.3%)	4 (5.4%)	5 (9.4%)		42 (10.5%)
**30s**	57 (31.5%)	20 (21.7%)	27 (36.5%)	26 (49.1%)		130 (32.5%)
**40s**	63 (34.8%)	55 (59.8%)	34 (45.9%)	18 (34%)		170 (42.5%)
**>50**	31 (17.1%)	14 (15.2%)	9 (12.2%)	4 (7.5%)		58 (14.5%)
**Primary health issue**					*p* < 0.01	
**Chronic diseases**	73 (40.3%)	56 (60.9%)	33 (44.6%)	8 (15.1%)		170 (42.5%)
**Malignant diseases**	42 (23.2%)	19 (20.7%)	30 (40.5%)	30 (56.6%)		121 (30.3%)
**Illness or disability caused by trauma**	36 (19.9%)	7 (7.6%)	7 (9.5%)	8 (15.1%)		58 (14.5%)
**Congenital or genetic diseases**	29 (16%)	9 (9.8%)	4 (5.4%)	7 (13.2%)		49 (12.3%)
**Rare diseases or others**	1 (0.6%)	1 (1.1%)	0	0		2 (0.5%)

**Table 3:** Analysis of survey item significance across the participant category groups: experience with health information management services.

No.	Questions	Disease Group	Health-Related Role	Age Group
**A5**	What methods do you usually use to record or manage your/your family’s main health problems?	* ^1^	-	-
**B1.**	Have you ever used commercially available or downloadable apps from the app store (or a built-in app on your smartphone, such as Samsung Health or Apple Health) or a health information measuring device (such as a smartwatch) to measure or record your blood pressure, blood sugar, step counts, physical activity, and heart rate?	***	***	**
**B2.**	Have you ever used a patient app suggested by your healthcare provider?	*	*	-
**B2a.**	→ (Y) Have you ever managed or searched for health information for yourself and your family using a patient app?	**	-	-
**B2b.**	→ (N) If you answered no, would you use a health management app service if your medical institution were to offer it?	***	*	**

^1^ Significant relationships are denoted by * (*p* < 0.05), ** (*p* < 0.01), and *** (*p* < 0.001).

**Table 4:** Analysis of the significance of survey item across participant category groups: attitude toward the use of PGHD.

No.	Questions	Disease Group	Health-Related Role	Age Group
**C1.**	To what extent do you agree to the collection of health-related information through health management apps or other health information measurement devices in combination with your medical information to make treatment plans and treatment-related decisions about you/your family?	-	-	-
**D1.**	To what extent do you agree with the use of your patient-generated health data (PGHD) for these research purposes?	-	-	-
**D1b.**	→ (Y) If you agree to the use of your or your family’s data for research purposes, to what extent would you like the data to be used?	-	** ^1^	-
**D1a.**	→ (N) What is the main reason you refuse or hesitate to share your health data for research purposes?	-	-	-
**D2.**	If you agree to the use of your or your family member’s PGHD for research purposes, do you wish to know the specific research purpose and duration? Alternatively, would you like to agree to a comprehensive research goal to improve healthcare?	**	**	-
**E1.**	To what extent do you agree with the use of combined medical data and PGHD for industrial/commercial purposes?	**	-	-
**F1.**	What are the most important steps or information you would like to know when consenting to the use of your/your family’s PGHD?	-	-	*

^1^ Significant relationships are denoted by * (*p* < 0.05), ** (*p* < 0.01)

**Table 5:** Comparison of reasons for selecting specific vs. comprehensive consent among respondents.

Rank	Reason for Selecting Specific Consent by Specific Research Purpose	Count	Reason for Selecting Comprehensive Consent	Count
**1**	Wish to know specific research purposes, contents, duration, etc.	75	For convenience	51
**2**	Wish to know how personal information is utilized in research	51	Think it will be helpful for research	24
**3**	Concerns regarding privacy breach, misuse, etc.	35	For saving time due to busyness	5
**4**	Think it will help healthcare professionals, research	13	Because consent terms are complicated, did not usually check content	5
**5**	Typically consider individual consent to be necessary	12	Trust in (research/institution/researcher)	4

**Table 6:** Additional responses and feedback on health information management methods and experiences with tethered PHR apps.

Item	Sub-Category	Responses
Methods used to record or manage your/your family’s main health problems (A5a)	Domestic social networking service (SNS) applications	Utilization of photo-centric SNS applications, blogs, and KakaoStory.
	Devices for Health Management	Management through devices capable of measuring blood glucose levels.
Best aspects of using tethered PHR for managing or accessing health information (B2a1)	Easier Access to Test Results	“It is easier to check test results compared with the use of printed documents.” “I can directly access test results without having to print documents or inquire at the hospital.”
	Self-Management of Health and Disease	“I can thoroughly review the test results.” “It is beneficial to be able to double-check the test results and appointment details.”
	Understanding Healthcare Provider’s Explanations	“Studying and reviewing the test results before appointments greatly helps in understanding, allowing for more in-depth questions to be asked to the attending physician.” “It is great to be able to inquire directly with the attending physician about any questions I have after reviewing the test results, enabling a better consultation experience.”
Frustrating Aspects of Patient Apps (B2a2)	Accumulation of Test Results	“I wish the test results could accumulate for over a year.”
	Confusion Over Information Accuracy	“I was rather confused because I did not know which information was correct.” “I felt disappointed when there were occasional readings that raised suspicion of measurement errors.” “It would be helpful if easy-to-understand terms were used.”

## Data Availability

The data underlying the results presented in this study are available from the Asan Medical Center Institutional Data Access/Ethics Committee. However, public disclosure of the data is not feasible as data sharing was not considered during the study design and IRB review. Researchers who meet the criteria for access to confidential data can contact the committee at irb@amc.seoul.kr or +82-02-3010-7166.

## Supplementary Materials

The following supporting information can be downloaded at: https://www.mdpi.com/article/10.3390/ijerph22071099/s1, Table S1: Complete Survey Instrument Including All Items and Response Options, Table S2: Significance Analysis of Survey Items by Disease Group; Table S3: Significance Analysis of Survey Items by Health-related Role of Respondent Groups; Table S4: Significance Analysis of Survey Items by Age Group.

## Abbreviations

The following abbreviations are used in this manuscript:

CG-adult	Caregivers of adult patients not falling into the previous categories
CG-elder	Caregivers of patients aged 70 or older or those unable to attend medical appointments alone
CG-minor	Caregivers of minor patients
PGHD	Patient-generated health data
PHRs	Personal Health Records
PROMs	Patient-reported outcome measures
Pt	Patients themselves
SNS	Social networking services
