# Supplementary material for: Perceptions and Willingness of Patients and Caregivers on the Utilization of Patient-Generated Health Data: A Cross-Sectional Survey
Source: Int J Environ Res Public Health. 2025 Jul 11;22(7):1099. doi: 10.3390/ijerph22071099 (PMC12294519; doi:10.3390/ijerph22071099)
Supplement: Supplementary file 1 [file ijerph-22-01099-s001.zip › Supplementary Materials (Table S1).pdf]

Supplementary Table 1. Complete Survey Instrument Including All Items and Response Options

Survey targeting Patients and Caregivers	
Classification	Survey content
Informed consent	1. I have been fully informed of the research details in accordance with the above, I understand the purpose and contents of the research, and I agree to participate in the survey.<br>① Agree (continue) ② Disagree (stop)
A. General information	A2. Gender<br>① Male ② Female<br><br>A3. Age<br>① 20s ② 30s ③ 40s ④ 50s and older<br><br>A4. Which of the following mainly apply to you when you use a healthcare provider? Please select the condition that most influences your response decision, even if it is not your current condition.<br>① Patients themselves.<br>② Caregivers of patients who are minors.<br>③ Caregivers of older patients aged 70 and above or older patients who face difficulty receiving hospital treatment alone.<br>④ Caregivers of adult patients who do not fall under categories 2 and 3.<br>A5. What is your/your family's **primary health** issue?<br>① Congenital or genetic diseases.<br>② Illness or disability caused by trauma, such as a fall or traffic accident.<br>③ Malignant diseases such as leukemia and other various types of cancer.<br>④ Chronic diseases such as hypertension, diabetes, cardiovascular/cerebrovascular diseases, chronic lung diseases (asthma/obstructive pulmonary disease), and dementia.<br>⑤ Unknown or other (please write the reason for choosing this response___).<br><br>A5a. What methods do you **usually** use to **record or manage your/your family's** main **health problems**?<br>① Handwritten records on paper, in notebooks, or in patient notebooks provided by the hospital.<br>② Unstructured electronic records, such as notepads and Word/excel files on your computer.<br>③ Basic functions of a cell phone, smartphone, or tablet (photo album or note-taking applications (apps)).<br>④ Apps dedicated to disease/health management on smartphones or tablets.<br>⑤ I do not usually record or manage them separately.<br>⑥ Other; please specify________________.
B. Experience with mobile healthcare and health information management services connected to medical institutions	B1. Have you ever **used** commercially available or downloadable apps from the **app** store (or a built-in app **on** your smartphone, such as Samsung Health or Apple Health) or a **health information measuring device (such as a smartwatch) to measure or record your** blood pressure, blood sugar, step counts, physical activity, and heart rate?<br>① Yes (☞ Go to Question B1a) ② No (☞ Go to Question B1d)<br><br>B1a. If you answered yes, how long have you been using it?<br>① Less than 1 month ② 1–3 months ③ 3–6 months ④ 6–12 months ⑤ More than 1 year<br><br>B1b. If you answered yes, what data did you measure? (Select all that apply)<br>① Physical activity (exercise, such as step counts).<br>② Blood sugar.<br>③ Blood pressure.<br>④ Heart Rate.<br>⑤ Sleep pattern.<br>⑥ Oxygen saturation.<br>⑦ Electrocardiogram (ECG).<br>⑧ Other; please specify_________.

	B1c. If you answered yes, how often do you use it?<br>① Daily<br>② Regularly, but not daily (e.g., once a week).<br>③ Occasionally (when I think of it)<br><br>B1d. If you are not currently using it, why did you stop?<br>① Because it was not helpful for my health management.<br>② Because it was annoying.<br>③ Because it was difficult to use.<br>④ Other; please specify__________.<br><br>B2. Have you ever used a **patient** app **provided by your healthcare organization?**<br>① Yes (☞ Go to Question B2a) ② No (☞ Go to Question B2b)<br><br>B2a Have you ever **managed** or searched for **health information** for yourself and your **family** using a **patient** app**?**<br>① Yes (☞ Go to Question B2a1) ② No (☞ Go to Question B2b)<br><br>B2a1. If you answered yes, what was the best part about using the app?<br>① It was helpful in the self-management of health and diseases.<br>② I have a better understanding of the healthcare provider's explanations or treatment plans.<br>③ It was easy and fun to use.<br>④ Provision of information on services other than medical treatment (such as scheduling appointments with healthcare providers and obtaining documents for submission to insurance companies).<br>⑤ Other, or provide any additional comments for the selected response<br><br>B2a2. If you answered yes, what was the most frustrating aspect of using the app?<br>① There was no helpful content.<br>② I was rather confused because I did not know which information was correct.<br>③ It was difficult to use.<br>④ Other, or provide any additional comments for the selected response.<br><br>B2b. If you answered no, would you use a health management app service if your medical institution were to offer it?<br>① Yes ② No
C. Consent for medical purpose use	C1. To what extent do you agree to the collection of health-related information through health management apps or other health information measurement devices in **combination with** your **medical information to make treatment plans** and **treatment-related decisions about you/**your family?<br>① Strongly disagree ② Disagree ③ Agree ④ Strongly agree
D1. Consent for research purpose use and method of consent	D1. Health-related information collected through health management apps or other health information measurement devices may be used for various scientific research purposes, such as predicting, treating, and preventing related diseases when combined with medical information. However, the results of these studies may not directly benefit you. To what extent do you agree with the use of **your patient-generated health data (PGHD) for** these **research purposes**?<br>① Strongly disagree ② Disagree ③ Agree ④ Strongly agree<br><br>D1a. What is the main reason you refuse or hesitate to share your health data for research purposes?<br>① Concerns regarding potential damage or disadvantage due to personal information leakage.<br>② Apprehension about not knowing where and how the information will be used or being unable to withdraw my consent.<br>③ Fear of not being properly recognized and rewarded for providing information<br>④ Apprehension regarding the trustworthiness of the place requesting information.<br>⑤ Other, please specify_________.

<table>
<tr><td></td><td>D1b. If you agree to the use of your or your family's data for research purposes, to what extent would you like the data to be used? The more potentially personally identifiable information is removed (processed), the less likely it is to be re-identified, which may also make the data less useful.
① Utilization of anonymous data that cannot identify individuals.
② Utilization of data that contains identifying information but are secure.

D2. If you agree to the use of your or your family member's PGHD for research purposes, do you want to know the specific research purpose and duration? Alternatively, would you like to agree to a comprehensive research goal to improve healthcare?
① Consent by specific research purpose: agree to each study (☞ Go to Question D2a).
② Comprehensive consent: agree all at once and do not ask again (☞ Go to Question D2b).

D2a. If you answered yes to “consent by specific research purpose,” why?

D2b. If you answered yes to “comprehensive consent,” why?</td></tr>
<tr><td>E. Consent to use for commercial purposes</td><td>E1. As explained above, the results of various studies that use PGHD can be used by healthcare providers to improve health outcomes and can be utilized in national health policy. However, they can also be used for industrial/commercial activities that seek profit by developing new drugs or programs. To what extent do you agree with the use of combined medical data and PGHD for industrial/commercial purposes?
① Strongly disagree (☞ Go to Question E1a)
② Disagree (☞ Go to Question E1a)
③ Agree (☞ Go to Question F1)
④ Strongly agree (☞ Go to Question F1)
E1a. Why do you disagree with its use for industrial/commercial purposes? (Rank the options by priority)
① Apprehension regarding damage or disadvantage due to personal information leakage.
② Apprehension about not knowing where and how the information is used.
③ Fear of being unable to withdraw my consent.
④ Fear of not feeling valued or not being rewarded for providing information.
⑤ Apprehension regarding the trustworthiness of the place requesting information.
⑥ Other; please specify______________.</td></tr>
<tr><td>F. Procedure for patient participation</td><td>F1. What are the most important steps or information you would like to know when consenting to the use of your/your family’s PGHD?
② How to withdraw consent.
③ Results of data analysis, research results, and implications.
④ Phone number to contact and information on the person in charge if you have questions or complaints about the research process.
⑤ Personal information protection and security issues related to data utilization.
⑥ Other; please specify__________.</td></tr>
</table>
