# Supplementary material for: Perceptions and Willingness of Patients and Caregivers on the Utilization of Patient-Generated Health Data: A Cross-Sectional Survey
Source: Int J Environ Res Public Health. 2025 Jul 11;22(7):1099. doi: 10.3390/ijerph22071099 (PMC12294519; doi:10.3390/ijerph22071099)
Supplement: Supplementary file 1 [file ijerph-22-01099-s001.zip › Supplementary Materials (Tables S2-S4).pdf]

Supplementary Table 2. Significance Analysis of Survey Items by Disease Group

No.	Questions	Congenital or genetic diseases (N=49)	Trauma (N=58)	Malignant diseases (N=121)	Chronic diseases (N=170)	Rare diseases or others (N=2)	*P*-value	Total (N=400)
A5a	*What methods do you usually use to record or manage your/your family's main health problems?*						***P* < .01	
	① Handwritten records on paper, in notebooks, or in patient notebooks provided by the hospital.	7 (14.3%)	5 (8.6%)	19 (15.7%)	16 (9.4%)	0		47 (11.8%)
	② Unstructured electronic records, such as notepads and Word/excel files on your computer.	5 (10.2%)	7 (12.1%)	10 (8.3%)	9 (5.3%)	0		31 (7.8%)
	③ Basic functions of a cell phone, smartphone, or tablet (photo album or note-taking applications (apps)).	21 (42.9%)	24 (41.4%)	51 (42.1%)	53 (31.2%)	2 (100%)		151 (37.8%)
	④ Apps dedicated to disease/health management on smartphones or tablets.	12 (24.5%)	8 (13.8%)	22 (18.2%)	71 (41.8%)	0		113 (28.2%)
	⑤ I do not usually record or manage them separately.	4 (8.2%)	14 (24.1%)	16 (13.2%)	19 (11.2%)	0		53 (13.2%)
	Other	0	0	3 (2.5%0	2 (1.2%)	0		5 (1.2%)
B1.	*Have you ever used commercially available or downloadable apps from the app store (or a built-in app on your smartphone, such as Samsung Health or Apple Health) or a health information measuring device (such as a smartwatch) to measure or record your blood pressure, blood sugar, step counts, physical activity, and heart rate?*						***P* < .01	
	① Yes	29 (59.2%)	22 (37.9%)	61 (50.4%)	134 (78.8%)	1 (50%)		247 (61.8%)
	② No	20 (40.8%)	36 (62.1%)	60 (49.6%)	36 (21.2%)	1 (50%)		153 (38.3%)
B1a.	*If you answered yes, how long have you been using it?*						0.06	
	① Less than 1 month	2 (6.9%)	3 (13.6%)	6 (9.8%)	14 (10.4%)	0		25 (10.1%)
	② 1-3 months	2 (6.9%)	2 (9.1%)	8 (13.1%)	14 (10.4%)	0		26 (10.5%)
	③ 3-6 months	6 (20.7%)	9 (40.9%)	10 (16.4%)	13 (9.7%)	0		38 (15.4%)
	④ 6-12 months	8 (27.6%)	6 (27.3%)	12 (19.7%)	28 (20.9%)	1 (100%)		55 (22.3%)
	⑤ More than 1 year	11 (37.9%)	2 (9.1%)	25 (41%)	65 (48.5%)	0		103 (41.7%)

B1c.	*If you answered yes, how often do you use it?*						0.23	
	① Daily	10 (34.5%)	12 (54.5%)	25 (41%)	77 (57.5%)	1 (100%)		125 (50.6%)
	② Regularly, but not daily (e.g., once a week).	15 (51.7%)	8 (36.4%)	24 (39.3%)	39 (29.1%)	0		86 (34.8%)
	③ Occasionally (when I think of it)	4 (13.8%)	2 (9.1%)	12 (19.7%)	18 (13.4%)	0		36 (14.6%)
B1d.	*If you are not currently using it, why did you stop? (Optional response item, N=160)*						**P* < .01	
	① Because it was not helpful for my health management.	5 (22.7%)	10 (27%)	12 (20%)	9 (22.5%)	0		36 (22.5%)
	② Because it was bothersome.	12 (54.5%)	19 (51.4%)	19 (31.7%)	19 (47.5%)	0		69 (43.1%)
	③ Because it was difficult to use.	5 (22.7%)	8 (21.6%)	25 (41.7%)	10 (25%)	0		48 (30%)
	④ Other	0	0	4 (6.7%)	2 (5%)	1 (100%)		7 (4.4%)
B2.	*Have you ever used a patient app provided by your healthcare organization?*						*0.04	
	① Yes	8 (16.3%)	6 (10.3%)	29 (24%)	20 (11.8%)	0		63 (15.8%)
	② No	41 (83.7%)	52 (89.7%)	92 (76%)	150 (88.2%)	2 (100%)		337 (84.3%)
B2a.	*Have you ever managed or searched for health information for yourself and your family using a patient app?*						**P* < .01	
	① Yes	5 (62.5%)	0	19 (65.5%)	17 (85%)	-		41 (65.1%)
	② No	3 (37.5%)	6 (100%)	10 (34.5%)	3 (15%)	-		22 (34.9%)
B2a1.	*If you answered yes, what was the best part about using the app?*						0.83	
	① It was helpful in the self-management of health and diseases.	4 (80%)	-	12 (63.2%)	10 (58.8%)	-		26 (63.4%)
	② I have a better understanding of the healthcare provider's explanations or treatment plans.	1 (20%)	-	2 (10.5%)	4 (23.5%)	-		7 (17.1%)
	③ It was easy and fun to use.	0	-	4 (21.1%)	2 (11.8%)	-		6 (14.6%)
	④ Provision of information on services other than medical treatment (such as scheduling appointments with healthcare providers and obtaining documents for submission to insurance companies).	0	-	1 (5.3%)	1 (5.9%)	-		2 (4.9%)
B2a2.	*If you answered yes, what was the most frustrating aspect of using the app?*						0.14	
	① There was no helpful content.	1 (20%)	-	0	1 (5.9%)	-		2 (4.9%)

	② I was rather confused because ③ I did not know which information was correct.	2 (40%)	-	7 (36.8%)	3 (17.6%)	-		12 (29.3%)
	④ It was difficult to use.	2 (40%)	-	6 (31.6%)	3 (17.6%)	-		11 (26.8%)
	⑤ Other	0	-	6 (31.6%)	10 (58.8%)	-		16 (39%)
B2b.	*If you answered no, would you use a health management app service if your medical institution were to offer it?*						***P* < .01	
	① Yes	35 (85.4%)	26 (50%)	58 (63%)	124 (82.7%)	2 (100%)		245 (72.7%)
	② No	6 (14.6%)	26 (50%)	34 (37%)	26 (17.3%)	0		92 (27.3%)
C1.	*To what extent do you agree to the collection of health-related information through health management apps or other health information measurement devices in combination with your medical information to make treatment plans and treatment-related decisions about you/your family?*						0.69	
	① Strongly disagree	0	1 (1.7%)	2 (1.7%)	2 (1.2%)	0		5 (1.3%)
	② Disagree	6 (12.2%)	10 (17.2%)	8 (6.6%)	14 (8.2%)	0		38 (9.5%)
	③ Agree	34 (69.4%)	36 (62.1%)	78 (64.5%)	113 (66.5%)	1 (50%)		262 (65.5%)
	④ Strongly agree	9 (18.4%)	11 (19%)	33 (27.3%)	41 (24.1%)	1 (50%)		95 (23.8%)
D1.	*Health-related information collected through health management apps or other health information measurement devices may be used for various scientific research purposes, such as predicting, treating, and preventing related diseases when combined with medical information. However, the results of these studies may not directly benefit you. To what extent do you agree with the use of your patient-generated health data (PGHD) for these research purposes?*						*0.02	
	① Strongly disagree	0	5 (8.6%)	3 (2.5%)	2 (1.2%)	0		10 (2.5%)
	② Disagree	7 (14.3%)	12 (20.7%)	10 (8.3%)	22 (12.9%)	0		51 (12.8%)
	③ Agree	28 (57.1%)	31 (53.7%)	68 (56.2%)	111 (65.3%)	1 (50%)		239 (59.8%)
	④ Strongly agree	14 (28.6%)	10 (17.2%)	40 (33.1%)	35 (20.6%)	1 (50%)		100 (25%)
D1b.	*If you agree to the use of your or your family's data for research purposes, to what extent would you like the data to be used? The*						0.87	

	more potentially personally identifiable information is removed (processed), the less likely it is to be re-identified, which may also make the data less useful.						
	① Utilization of anonymous data that cannot identify individuals.	23 (56.1%)	20 (48.8%)	61 (56.5%)	85 (58.2%)	1 (50%)		190 (56.2%)
	② Utilization of data that contains identifying information but are secure.	18 (43.9%)	21 (51.2%)	47 (43.5%)	61 (41.8%)	1 (50%)		148 (43.8%)
D1a.	*What is the main reason you refuse or hesitate to share your health data for research purposes?*						0.44	
	① Concerns regarding potential damage or disadvantage due to personal information leakage.	5 (71.4%)	10 (58.8%)	5 (38.5%)	9 (37.5%)	-		29 (47.5%)
	② Apprehension about not knowing where and how the information will be used or being unable to withdraw my consent.	1 (14.3%)	5 (29.4%)	5 (38.5%)	5 (20.8%)	-		16 (26.2%)
	③ Fear of not being properly recognized and rewarded for providing information	0	2 (11.8%)	2 (15.4%)	6 (25%)	-		10 (16.4%)
	④ Apprehension regarding the trustworthiness of the place requesting information.	1 (14.3%)	0	1 (7.7%)	4 (16.7%)	-		6 (9.8%)
D2.	*If you agree to the use of your or your family member's PGHD for research purposes, do you want to know the specific research purpose and duration? Alternatively, would you like to agree to a comprehensive research goal to improve healthcare?*						*0.01	
	① Consent by specific research purpose: agree to each study	28 (57.1%)	36 (62.1%0	57 (47.1%)	117 (68.8%)	1 (50%)		239 (59.8%)
	② Comprehensive consent: agree all at once and do not ask again	21 (42.9%)	22 (37.9%)	64 (52.9%)	53 (31.2%)	1 (50%)		161 (40.3%)
E1.	*As explained above, the results of various studies that use PGHD can be used by healthcare providers to improve health outcomes and can be utilized in national health policy. However, they can also be used for industrial/commercial activities that seek profit by developing new drugs or programs. To what extent do you agree with the use of combined medical data and PGHD for industrial/commercial purposes?*						*0.04	
	① Strongly disagree	1 (2%)	7 (12.1%)	7 (5.8%)	12 (7.1%)	0		27 (6.8%)
	② Disagree	6 (12.2%)	14	16 (13.2%)	37	1		74

			(24.1%)		(21.8%)	(50%)		(18.5%)
	③ Agree	32 (65.3%)	26 (44.8%)	71 (58.7%)	105 (61.8%)	1 (50%)		235 (58.8%)
	④ Strongly agree	10 (20.4%)	11 (19%)	27 (22.3%)	16 (9.4%)	0		64 (16%)
F1.	*What are the most important steps or information you would like to know when consenting to the use of your/your family's PGHD?*						***P* < .01	
	① How to withdraw consent.	5 (10.2%)	14 (24.1%)	21 (17.4%)	18 (10.6%)	0		27 (6.8%)
	② Results of data analysis, research results, and implications.	28 (57.1%)	12 (20.7%)	53 (43.8%)	71 (41.8%)	2 (100%)		74 (18.5%)
	③ Phone number to contact and information on the person in charge if you have questions or complaints about the research process.	13 (26.5%)	13 (22.4%)	18 (14.9%)	26 (15.3%)	0		235 (58.8%)
	④ Personal information protection and security issues related to data utilization.	3 (6.1%)	19 (32.8%)	29 (24%)	52 (30.6%)	0		64 (16%)
	⑤ Other	0	0	0	3 (1.8%)	0		0

Notes: 1) B2a and D1a were optional items, and responses from the "Rare diseases or others" group (N=2) were excluded from the analysis due to no responses. 2) B2a1 and B2a2 were optional items, and responses from both the "Rare diseases or others" and "Trauma" groups were excluded from the analysis due to no responses. 3) One response from the "Congenital or genetic diseases" group is missing in item D1b.

Supplementary Table 3. Significance Analysis of Survey Items by Health-related Role of Respondent Groups

No.	Questions	Pt (N=181)	CG-minor (N=92)	CG-elder (N=74)	CG-adult (N=53)	*P*-value	Total (N=400)
A5a	*What methods do you usually use to record or manage your/your family's main health problems?*					0.07	
	① Handwritten records on paper, in notebooks, or in patient notebooks provided by the hospital.	20 (11%)	7 (7.6%)	10 (13.5%)	10 (18.9%)		47 (11.8%)
	② Unstructured electronic records, such as notepads and Word/excel files on your computer.	13 (7.2%)	5 (5.4%)	6 (8.1%)	7 (13.2%)		31 (7.8%)
	③ Basic functions of a cell phone, smartphone, or tablet (photo album or note-taking applications (apps)).	74 (40.9%)	35 (38%)	26 (35.1%)	16 (30.2%)		151 (37.8%)
	④ Apps dedicated to disease/health management on smartphones or tablets.	46 (25.4%)	36 (39.1%)	24 (32.4%)	7 (13.2%)		113 (28.3%)
	⑤ I do not usually record or manage them separately.	25 (13.8%)	8 (8.7%)	8 (10.8%)	12 (22.6%)		53 (13.3%)
	Other	3 (1.7%)	1 (1.1%)	0	1 (1.9%)		5 (1.3%)
B1.	*Have you ever used commercially available or downloadable apps from the app store (or a built-in app on your smartphone, such as Samsung Health or Apple Health) or a health information measuring device (such as a smartwatch) to measure or record your blood pressure, blood sugar, step counts, physical activity, and heart rate?*					***P* < .01	
	① Yes	121 (66.9%)	69 (75%)	35 (47.3%)	22 (41.5%)		247 (61.8%)
	② No	60 (33.1%)	23 (25%)	39 (52.7%)	31 (58.5%)		153 (38.3%)
B1a.	*If you answered yes, how long have you been using it?*					*0.02	
	① Less than 1 month	13 (10.7%)	9 (13%)	3 (8.6%)	0		25 (10.1%)
	② 1-3 months	12 (9.9%)	8 (11.6%)	2 (5.7%)	4 (18.2%)		26 (10.5%)
	③ 3-6 months	20 (16.5%)	7 (10.1%)	3 (8.6%)	8 (36.4%)		38 (15.4%)
	④ 6-12 months	33 (27.4%)	12 (17.4%)	5 (14.3%)	5 (22.7%)		55 (22.3%)
	⑤ More than 1 year	43 (35.5%)	33 (47.8%)	22 (62.9%)	5 (22.7%)		103 (41.7%)
B1c.	*If you answered yes, how often do you use it?*					*0.01	
	① Daily	63 (52.1%)	44 (63.8%)	11 (31.4%)	7 (31.8%)		125 (50.6%)

	② Regularly, but not daily (e.g., once a week).	45 (37.2%)	13 (18.8%)	18 (51.4%)	10 (45.5%)		86 (34.8%)
	③ Occasionally (when I think of it)	13 (10.7%)	12 (17.4%)	6 (17.1%)	5 (22.7%)		36 (14.6%)
B1d.	*If you are not currently using it, why did you stop? (Optional response item, N=160)*					0.75	
	① Because it was not helpful for my health management.	13 (20.6%)	8 (33.3%)	8 (20%)	7 (21.2%)		36 (22.5%)
	② Because it was bothersome.	32 (50.8%)	9 (37.5%)	14 (35%)	14 (42.4%)		69 (43.1%)
	③ Because it was difficult to use.	16 (25.4%)	6 (25%)	15 (37.5%)	11 (33.3%)		48 (30%)
	④ Other	2 (3.2%)	1 (4.2%)	3 (7.5%)	1 (3%)		7 (4.4%)
B2.	*Have you ever used a patient app provided by your healthcare organization?*					*0.01	
	① Yes	37 (20.4%)	17 (18.5%)	6 (8.1%)	3 (5.7%)		63 (15.8%)
	② No	144 (79.6%)	75 (81.5%)	68 (91.9%)	50 (94.3%)		337 (84.3%)
B2a.	*Have you ever managed or searched for health information for yourself and your family using a patient app?*					0.46	
	① Yes	23 (62.2%)	12 (70.6%)	5 (83.3%)	1 (33.3%)		41 (65.1%)
	② No	14 (37.8%)	5 (29.4%)	1 (16.7%)	2 (66.7%)		22 (34.9%)
B2a 1.	*If you answered yes, what was the best part about using the app?*					0.69	
	① It was helpful in the self-management of health and diseases.	13 (56.5%)	8 (66.7%)	4 (80%)	1 (100%)		26 (63.4%)
	② I have a better understanding of the healthcare provider's explanations or treatment plans.	4 (17.4%)	3 (25%)	0	0		7 (17.1%)
	③ It was easy and fun to use.	5 (21.7%)	0	1 (20%)	0		6 (14.6%)
	④ Provision of information on services other than medical treatment (such as scheduling appointments with healthcare providers and obtaining documents for submission to insurance companies).	1 (4.3%)	1 (8.3%)	0	0		2 (4.9%)
B2a 2.	*If you answered yes, what was the most frustrating aspect of using the app?*					0.71	
	① There was no helpful content.	1 (4.3%)	1 (8.3%)	0	0		2 (4.9%)
	② I was rather confused because ③ I did not know which information was correct.	8 (34.8%)	4 (33.3%)	0	0		12 (29.3%)
	④ It was difficult to use.	5 (21.7%)	3 (25%)	3 (60%)	0		11 (26.8%)
	⑤ Other	9 (39.1%)	4 (33.3%)	2 (40%)	1 (100%)		16 (39%)

B2b.	*If you answered no, would you use a health management app service if your medical institution were to offer it?*					*0.02	
	① Yes	100 (69.4%)	65 (86.7%)	48 (70.6%)	32 (64%)		245 (72.7%)
	② No	44 (30.6%)	10 (13.3%)	20 (29.4%)	18 (36%)		92 (27.3%)
C1.	*To what extent do you agree to the collection of health-related information through health management apps or other health information measurement devices in combination with your medical information to make treatment plans and treatment-related decisions about you/your family?*					0.18	
	① Strongly disagree	3 (1.7%)	1 (1.1%)	0	1 (1.9%)		5 (1.3%)
	② Disagree	18 (9.9%)	8 (8.7%)	9 (12.2%)	3 (5.7%)		38 (9.5%)
	③ Agree	113 (62.4%)	54 (58.7%)	53 (71.6%)	42 (79.2%)		262 (65.5%)
	④ Strongly agree	47 (26%)	29 (31.5%)	12 (16.2%)	7 (13.2%)		95 (23.8%)
D1.	*Health-related information collected through health management apps or other health information measurement devices may be used for various scientific research purposes, such as predicting, treating, and preventing related diseases when combined with medical information. However, the results of these studies may not directly benefit you. To what extent do you agree with the use of your patient-generated health data (PGHD) for these research purposes?*					0.75	
	① Strongly disagree	5 (2.8%)	3 (3.3%)	0	2 (3.8%)		10 (2.5%)
	② Disagree	27 (14.9%)	8 (8.7%)	9 (12.2%)	7 (13.2%)		51 (12.8%)
	③ Agree	102 (56.4%)	56 (60.9%)	49 (66.2%)	32 (60.4%)		239 (59.8%)
	④ Strongly agree	47 (26%)	25 (27.2%)	16 (21.6%)	12 (22.6%)		100 (25%)
D1b.	*If you agree to the use of your or your family's data for research purposes, to what extent would you like the data to be used? The more potentially personally identifiable information is removed (processed), the less likely it is to be re-identified, which may also make the data less useful.*					*0.01	
	① Utilization of anonymous data that cannot identify individuals.	83 (55.7%)	34 (42%)	42 (64.6%)	31 (72.1%)		190 (56.2%)

	② Utilization of data that contains identifying information but are secure.	66 (44.3%)	47 (58%)	23 (35.4%)	12 (27.9%)		148 (43.8%)
D1a.	*What is the main reason you refuse or hesitate to share your health data for research purposes?*					0.44	
	① Concerns regarding potential damage or disadvantage due to personal information leakage.	14 (43.8%)	4 (36.4%)	4 (44.4%)	7 (77.8%)		29 (47.5%)
	② Apprehension about not knowing where and how the information will be used or being unable to withdraw my consent.	10 (31.3%)	2 (18.2%)	4 (44.4%)	0		16 (26.2%)
	③ Fear of not being properly recognized and rewarded for providing information	5(15.6%)	3 (27.3%)	1 (11.1%)	1 (11.1%)		10 (16.4%)
	④ Apprehension regarding the trustworthiness of the place requesting information.	3 (9.4%)	2 (18.2%)	0	1 (11.1%)		6 (9.8%)
D2.	*If you agree to the use of your or your family member's PGHD for research purposes, do you want to know the specific research purpose and duration? Alternatively, would you like to agree to a comprehensive research goal to improve healthcare?*					*0.01	
	① Consent by specific research purpose: agree to each study	115 (63.5%)	63 (68.5%)	33 (44.6%)	28 (52.8%)		239 (59.8%)
	② Comprehensive consent: agree all at once and do not ask again	66 (36.5%)	29 (31.5%)	41 (55.4%)	25 (47.2%)		161 (40.3%)
E1.	*As explained above, the results of various studies that use PGHD can be used by healthcare providers to improve health outcomes and can be utilized in national health policy. However, they can also be used for industrial/commercial activities that seek profit by developing new drugs or programs. To what extent do you agree with the use of combined medical data and PGHD for industrial/commercial purposes?*					0.12	
	① Strongly disagree	9 (5%)	7 (7.6%)	3 (4.1%)	8 (15.1%)		27 (6.8%)
	② Disagree	39 (21.5%)	18 (19.6%)	9 (12.2%)	8 (15.1%)		74 (18.5%)
	③ Agree	100 (55.2%)	52 (56.5%)	51 (68.9%)	32 (60.4%)		235 (58.8%)
	④ Strongly agree	33 (18.2%)	15 (16.3%)	11 (14.9%)	5 (9.4%)		64 (16%)
F1.	*What are the most important steps or information you would like to know when consenting to the use*					0.28	

of your/your family's PGHD?					
① How to withdraw consent.	26 (14.4%)	8 (8.7%)	14 (18.9%)	10 (18.9%)	58 (14.5%)
② Results of data analysis, research results, and implications.	80 (44.2%)	41 (44.6%)	25 (33.8%)	20 (37.7%)	166 (41.5%)
③ Phone number to contact and information on the person in charge if you have questions or complaints about the research process.	35 (19.3%)	11 (12%)	14 (18.9%)	10 (18.9%)	70 (17.5%)
④ Personal information protection and security issues related to data utilization.	39 (21.5%)	30 (32.6%)	21 (28.4%)	13 (24.5%)	103 (25.8%)
⑤ Other	1 (0.6%)	2 (2.2%)	0	0	3 (0.8%)

Note: One response from the "CG-adult" group is missing in item D1b.

Supplementary Table 4. Significance Analysis of Survey Items by Age Group

No.	Questions	20s (N=42)	30s (N=130)	40s (N=170)	50s and older (N=58)	*P*-value	Total (N=400)
A5a	*What methods do you usually use to record or manage your/your family's main health problems?*					0.28	
	① Handwritten records on paper, in notebooks, or in patient notebooks provided by the hospital.	2 (4.8%)	20 (15.4%)	14 (8.2%)	11 (19%)		47 (11.8%)
	② Unstructured electronic records, such as notepads and Word/excel files on your computer.	1 (2.4%)	12 (9.2%)	13 (7.6%)	5 (8.6%)		31 (7.8%)
	③ Basic functions of a cell phone, smartphone, or tablet (photo album or note-taking applications (apps)).	18 (42.9%)	42 (32.3%)	69 (40.6%)	22 (37.9%)		151 (37.8%)
	④ Apps dedicated to disease/health management on smartphones or tablets.	12 (28.6%)	39 (30%)	49 (28.8%)	13 (22.4%)		113 (28.3%)
	⑤ I do not usually record or manage them separately.	9 (21.4%)	16 (12.3%)	21 (12.4%)	7 (12.1%)		53 (13.3%)
	Other	0	1 (0.8%)	4 (2.4%)	0		5 (1.3%)
B1.	*Have you ever used commercially available or downloadable apps from the app store (or a built-in app on your smartphone, such as Samsung Health or Apple Health) or a health information measuring device (such as a smartwatch) to measure or record your blood pressure, blood sugar, step counts, physical activity, and heart rate?*					*0.01	
	① Yes	22 (52.4%)	69 (53.1%)	120 (70.6%)	36 (62.1%)		247 (61.8%)
	② No	20 (47.6%)	61 (46.9%)	50 (29.4%)	22 (37.9%)		153 (38.3%)
B1a.	*If you answered yes, how long have you been using it?*					0.10	
	① Less than 1 month	1 (4.5%)	9 (13%)	13 (10.8%)	2 (5.6%)		25 (10.1%)
	② 1-3 months	1 (4.5%)	13 (18.8%)	10 (8.3%)	2 (5.6%)		26 (10.5%)
	③ 3-6 months	3 (13.6%)	12 (17.4%)	20 (16.7%)	3 (8.3%)		38 (15.4%)
	④ 6-12 months	5 (22.7%)	9 (13%)	33 (27.5%)	8 (22.2%)		55 (22.3%)
	⑤ More than 1 year	12 (54.%)	26 (37.7%)	44 (36.7%)	21 (58.3%)		103 (41.7%)
B1c.	*If you answered yes, how often do you use it?*					0.39	
	① Daily	11 (50%)	36 (52.2%)	56 (46.7%)	22 (61.1%)		125 (50.6%)

	② Regularly, but not daily (e.g., once a week).	7 (31.8%)	21 (30.4%)	50 (41.7%)	8 (22.2%)		86 (34.8%)
	③ Occasionally (when I think of it)	4 (18.2%)	12 (17.4%)	14 (11.7%)	6 (16.7%)		36 (14.6%)
B1d.	*If you are not currently using it, why did you stop? (Optional response item, N=160)*					*0.01	
	① Because it was not helpful for my health management.	7 (35%)	12 (19%)	9 (17.3%)	8 (32%)		36 (22.5%)
	② Because it was bothersome.	10 (50%)	35 (55.6%)	18 (34.6%)	6 (24%)		69 (43.1%)
	③ Because it was difficult to use.	3 (15%)	13 (20.6%)	24 (46.2%)	8 (32%)		48 (30%)
	④ Other	0	3 (4.8%)	1 (1.9%)	3 (12%)		7 (4.4%)
B2.	*Have you ever used a patient app provided by your healthcare organization?*					0.51	
	① Yes	4 (9.5%)	21 (16.2%)	26 (15.3%)	12 (20.7%)		63 (15.8%)
	② No	38 (90.5%)	109 (83.8%)	144 (84.7%)	46 (79.3%)		337 (84.3%)
B2a.	*Have you ever managed or searched for health information for yourself and your family using a patient app?*					0.26	
	① Yes	2 (50%)	15 (71.4%)	14 (53.8%)	10 (83.3%)		41 (65.1%)
	② No	2 (50%)	6 (28.6%)	12 (46.2%)	2 (16.7%)		22 (34.9%)
B2a1.	*If you answered yes, what was the best part about using the app?*					0.20	
	① It was helpful in the self-management of health and diseases.	1 (50%)	9 (60%)	9 (64.3%)	7 (70%)		26 (63.4%)
	② I have a better understanding of the healthcare provider's explanations or treatment plans.	0	1 (6.7%)	5 (35.7%)	1 (10%)		7 (17.1%)
	③ It was easy and fun to use.	1 (50%)	3 (20%)	0	2 (20%)		6 (14.6%)
	④ Provision of information on services other than medical treatment (such as scheduling appointments with healthcare providers and obtaining documents for submission to insurance companies).	0	2 (13.3%)	0	0		2 (4.9%)
B2a2.	*If you answered yes, what was the most frustrating aspect of using the app?*					0.98	
	① There was no helpful content.	0	1 (6.7%)	1 (7.1%)	0		2 (4.9%)
	② I was rather confused because ③ I did not know which information was correct.	0	5 (33.3%)	4 (28.6%)	3 (30%)		12 (29.3%)
	④ It was difficult to use.	1 (50%)	4 (26.7%)	4 (28.6%)	2 (20%)		11 (26.8%)
	⑤ Other	1 (50%)	5 (33.3%)	5 (35.7%)	5 (50%)		16 (39%0

B2b.	*If you answered no, would you use a health management app service if your medical institution were to offer it?*					*$**P$* < .01	
	① Yes	23 (60.5%)	70 (64.2%)	118 (81.9%)	34 (73.9%)		245 (72.7%)
	② No	15 (39.5%)	39 (35.8%)	26 (18.1%)	12 (26.1%)		92 (27.3%)
C1.	*To what extent do you agree to the collection of health-related information through health management apps or other health information measurement devices in combination with your medical information to make treatment plans and treatment-related decisions about you/your family?*					0.43	
	① Strongly disagree	1 (2.4%)	1 (0.8%)	2 (1.2%)	1 (1.7%)		5 (1.3%)
	② Disagree	8 (19%)	14 (10.8%)	11 (6.5%)	5 (8.6%)		38 (9.5%)
	③ Agree	27 (64.3%)	81 (62.3%)	116 (68.2%)	38 (65.5%)		262 (65.5%)
	④ Strongly agree	6 (14.3%)	34 (26.2%)	41 (24.1%)	14 (24.1%)		95 (23.8%)
D1.	*Health-related information collected through health management apps or other health information measurement devices may be used for various scientific research purposes, such as predicting, treating, and preventing related diseases when combined with medical information. However, the results of these studies may not directly benefit you. To what extent do you agree with the use of your patient-generated health data (PGHD) for these research purposes?*					*$**P$* < .01	
	① Strongly disagree	3 (7.1%)	3 (2.3%)	1 (0.6%)	3 (5.2%)		10 (2.5%)
	② Disagree	13 (31%)	14 (10.8%)	19 (11.2%)	5 (8.6%)		51 (12.8%)
	③ Agree	18 (42.9%)	81 (62.3%)	102 (60%)	38 (65.5%)		239 (59.8%)
	④ Strongly agree	8 (19%)	32 (24.6%)	48 (28.2%)	12 (20.7%)		100 (25%)
D1b.	*If you agree to the use of your or your family's data for research purposes, to what extent would you like the data to be used? The more potentially personally identifiable information is removed (processed), the less likely it is to be re-identified, which may also make the data less useful.*					0.30	
	① Utilization of anonymous data that cannot identify individuals.	19 (73.1%)	64 (57.1%)	80 (53.3%)	27 (54%)		190 (56.2%)

	② Utilization of data that contains identifying information but are secure.	7 (26.9%)	48 (42.9%)	70 (46.7%)	23 (46%)		148 (43.8%)
D1a.	*What is the main reason you refuse or hesitate to share your health data for research purposes?*					0.46	
	① Concerns regarding potential damage or disadvantage due to personal information leakage.	10 (62.5%)	7 (41.2%)	11 (55%)	1 (12.5%)		29 (47.5%)
	② Apprehension about not knowing where and how the information will be used or being unable to withdraw my consent.	3 (18.8%)	4 (23.5%)	6 (30%)	3 (37.5%)		16 (26.2%)
	③ Fear of not being properly recognized and rewarded for providing information	2 (12.5%)	3 (17.6%)	2 (10%)	3 (37.5%)		10 (16.4%)
	④ Apprehension regarding the trustworthiness of the place requesting information.	1 (6.3%)	3 (17.6%)	1 (5%)	1 (12.5%)		6 (9.8%)
D2.	*If you agree to the use of your or your family member's PGHD for research purposes, do you want to know the specific research purpose and duration? Alternatively, would you like to agree to a comprehensive research goal to improve healthcare?*					0.27	
	① Consent by specific research purpose: agree to each study	30 (71.4%)	71 (54.6%)	102 (60%)	36 (62.1%)		239 (59.8%)
	② Comprehensive consent: agree all at once and do not ask again	12 (28.6%)	59 (45.4%)	68 (40%)	22 (37.9%)		161 (40.3%)
E1.	*As explained above, the results of various studies that use PGHD can be used by healthcare providers to improve health outcomes and can be utilized in national health policy. However, they can also be used for industrial/commercial activities that seek profit by developing new drugs or programs. To what extent do you agree with the use of combined medical data and PGHD for industrial/commercial purposes?*					0.06	
	① Strongly disagree	5 (11.9%)	9 (6.9%)	10 (5.9%)	3 (5.2%)		27 (6.8%)
	② Disagree	15 (35.7%)	19 (14.6%)	26 (15.3%)	14 (24.1%)		74 (18.5%)
	③ Agree	19 (45.2%)	78 (60%)	106 (62.4%)	32 (55.2%)		235 (58.8%)
	④ Strongly agree	3 (7.1%)	24 (18.5%)	28 (16.5%)	9 (15.5%)		64 (16%)
F1.	*What are the most important steps or information you would like to know when consenting to the use*					0.21	

of your/your family's PGHD?					
① How to withdraw consent.	6 (14.3%)	23 (17.7%)	26 (15.3%)	3 (5.2%)	58 (14.5%)
② Results of data analysis, research results, and implications.	14 (33.3%)	53 (40.8%)	76 (44.7%)	23 (39.7%)	166 (41.5%)
③ Phone number to contact and information on the person in charge if you have questions or complaints about the research process.	7 (16.7%)	23 (17.7%)	31 (18.2%)	9 (15.5%)	70 (17.5%)
④ Personal information protection and security issues related to data utilization.	15 (35.7%)	29 (22.3%)	36 (21.2%)	23 (39.7%)	103 (25.8%)
⑤ Other	0	2 (1.5%)	1 (0.6%)	0	3 (0.8%)

Note: One response from the "30s" group is missing in item D1b.
